# Operative and non-operative management for intestinal emergencies: findings from a single-centre retrospective cohort study

**DOI:** 10.1308/rcsann.2023.0093

**Published:** 2023-12-01

**Authors:** AR Darbyshire, I Kostakis, P Meredith, C Kovacs, D Prytherch, J Briggs, SKC Toh

**Affiliations:** ^1^ Portsmouth Hospitals University NHS Trust, UK; ^2^ Centre for Healthcare Modelling and Informatics, University of Portsmouth, UK

**Keywords:** Emergency, Laparotomy, Laparoscopy, EWS, Frailty

## Abstract

**Background:**

Patients with an intestinal emergency who do not have surgery are poorly characterised. This study used electronic healthcare records to provide a rapid insight into the number of patients admitted with an intestinal emergency and compare short-term outcomes for non-operative and operative management.

**Methods:**

A single-centre retrospective cohort study was conducted at a tertiary NHS hospital (from 1 December 2013 to 31 January 2020). Patients were identified using diagnosis codes for intestinal emergencies, based on the inclusion criteria for the National Emergency Laparotomy Audit. Relevant data were extracted from electronic healthcare records (*n*=3,997).

**Results:**

Nearly half of patients admitted with an intestinal emergency received nonoperative management (43.7%). Of those who underwent surgery, 63.7% were started laparoscopically. The non-operative group had a shorter hospital stay (median: 5.4 days vs 8.2 days [started laparoscopically] or 16.8 days [started open]) and fewer unintended intensive care admissions than the surgical group (2.4% vs 8.7% [started laparoscopically] 21.1% [started open]). However, 30-day mortality for non-operative treatment was double that for surgery (22.4% vs 10.1%). The 30-day mortality rate was found to be even higher for non-operative management (50.3%) compared with surgery (19.5%) in a sub-analysis of patients with admission National Early Warning Score ≥4 (*n*=683).

**Conclusion:**

The proportion of patients with intestinal emergencies who do not have surgery is greater than expected, and it appears that many respond well to non-operative treatment. However, 30-day mortality for non-operative management was high, and the low number of admissions to intensive care suggests that major invasive treatment was not appropriate for most in this group.

## Introduction

Intestinal emergencies are a diverse group of conditions that often require surgery.^1^ Some of these require urgent transfer to theatre, such as perforated peptic ulcers and mesenteric ischaemia.^2,3^ However, others, such as adhesional small bowel obstruction and complex diverticulitis, are readily treated by non-operative management, with surgery reserved as second-line treatment.^4,5^ Emergency bowel surgery is now the subject of national audits that monitor outcomes against evidence-based standards of care.^1,6^ However, a group for which there are few data is patients with an intestinal emergency who may need (but do not have) emergency bowel surgery. This is partly because of the presumption, particularly in cases where urgent surgery is required, that survival is unlikely.

A single-centre cohort study in Scotland has been first to investigate this topic, with revealing findings. It identified that a surprising 32% (*n*=100 of 314) of patients with an intestinal emergency were declined emergency bowel surgery, with the reason in 74% of cases cited as being due to ‘poor fitness’.^7^ The 30-day mortality rate for patients managed non-operatively was much higher than that for those who underwent surgery (63% vs 13%). Interestingly, risk-adjusted analysis suggested that 30-day mortality for this group would have been considerably lower (30–40%) if they had undergone surgery.^7^ While these findings are not definitive, they merit further investigation.

Gathering large amounts of data on patients prospectively can be time consuming and expensive. To quickly get a better understanding of this cohort of patients receiving non-operative management of intestinal emergencies, we decided to use a retrospective approach utilising electronic healthcare records (EHRs). The aim of this study was to use EHRs to identify all patients admitted to hospital with an intestinal emergency and compare short-term outcomes for those who were treated with emergency bowel surgery with outcomes for those who were not.

## Methods

This study is reported in line with the Reporting of studies Conducted using Observational Routinely Collected Data (RECORD) statement (checklist in Supplementary Appendix 1).^8^

### Study design and setting

This is a single-centre retrospective cohort study conducted at Portsmouth Hospitals University NHS Trust (PHU) using data from existing electronic health records between December 2013 and January 2020.

### Outcomes

The primary outcome is the number of admissions of patients with intestinal emergencies and the treatment received, be that open or laparoscopic surgery or non-operative management. The secondary outcomes are the rate of conversion to open surgery, 30-day, in-hospital and 1-year mortality, unintended admission to intensive care unit (ICU), length of hospital stay and readmission within 1 year. Outcomes have been compared between non-operative and operative groups, with the operative group divided into open and laparoscopic surgery.

### Participants

The study population was identified using the 10th revision of the International Statistical Classification of Diseases and Related Health Problems (ICD-10) diagnosis codes for intestinal emergencies. Patients aged 16 years or older who were admitted with an intestinal emergency were eligible for inclusion. Eligible participants must also have had a full set of vital signs and routine blood tests recorded during admission. Maternity admissions were excluded.

To identify the study population, a comprehensive list of ICD-10 codes was selected based on the inclusion criteria of National Emergency Laparotomy Audit (NELA) and clinical expertise. We started with the broadest possible approach to avoid accidentally excluding relevant episodes. This identified far more patients than anticipated and the list was then extensively refined using an iterative process of trial data extractions. Ultimately, ICD-10 codes for only the clear surgical conditions (from the NELA inclusion criteria) as the primary diagnosis for that admission were used.

### Data source

All data on patient demographics, admissions, diagnosis and procedure codes, vital signs and operating theatre and ICU data were extracted from existing EHRs at PHU. The local NELA dataset for the study period was downloaded from the NELA servers.

### Variables

National early warning score (NEWS) values were calculated from patient vital signs.^9^ The score from the first available vital signs observation during an admission was classified as admission NEWS, unless this was recorded after surgery.

Continuous variables were not dichotomised or grouped for analysis. The only exceptions were high NEWS observations, which were grouped together for visualisation in plots.

Operative approach was determined using OPCS-4 codes and cross-referenced with the NELA dataset. We opted to define this on an intention-to-treat basis, so all cases considered as open were started as a laparotomy. All laparoscopic cases were started as such and include laparoscopically assisted and cases converted to open. When calculating the conversion to open rate, laparoscopically assisted cases were considered as converted to open, as the size/nature of the incision used is not available.

Unintended admission to ICU (UICU) was differentiated from a planned postoperative admission to ICU if it occurred more than 24 hours after the beginning of surgery.

### Missing data

The only missing data items were vital signs/NEWS scores, but this was rare (1.15%). Missingness appeared to be random over time. Patient episodes with missing admission NEWS were therefore omitted from any analysis involving NEWS.

### Sample size

The anticipated sample size was calculated using the known size of the NELA dataset for the study period (*n*=1,500) and the assumption that an additional 30% of cases who were managed non-operatively would be identified, based on the findings of McIlveen *et al.*^7^ A sample size of approximately 2,000 cases was felt to be sufficient for this single-centre exploratory study, and the largest published to date.

### Statistical analysis

Data were summarised with descriptive statistics including counts and proportions, mean (±SD) and median (IQR) as appropriate.

We investigated the distribution of admission NEWS values, expressed as a proportion of total number of observations, by plotting them against 30-day mortality for each possible value. This allows the thresholds of risk for each possible score to be visualised.

Data analysis was performed in R Studio: R Foundation for Statistical Computing 2020 (Vienna, Austria).

### Bias

In this exploratory retrospective study, we provide only descriptive statistics and have not performed a comparative statistical analysis. We recognise that there will be selection bias influencing what treatment patients have received, and have interpreted the results accordingly. We have not attempted to control for confounding factors in this paper.

## Results

The final study cohort included 3,997 patients. Patient demographics and outcomes are summarised in Table 1. Just under half of patients received non-operative management (43.7%), which is higher than anticipated. Of patients who underwent surgery, laparoscopy was the favoured operative approach (63.7%). The rate of conversion to open surgery for emergency laparoscopy was low (21.4%, 306 of 1,432).

**Table 1:** Summary of demographic and admission data and outcomes for patients having open surgery, laparoscopy and non-operative treatment

	Open	Laparoscopic	Non-operative
Admissions, *n*	817	1,432	1,748
Age, years, median (IQR)	71 (59–80)	61 (44.8–74)	72 (58–82)
Female, *n* (%)	425 (52.0)	813 (56.8)	945 (54.1)
Type of admission			
Elective, *n* (%)	54 (6.6)	46 (3.2)	0 (0.0)
Emergency, *n* (%)	763 (93.4)	1,386 (96.8)	1,748 (100)
Admission specialty group			
General Medicine, *n* (%)	149 (18.2)	168 (11.7)	628 (35.9)
General Surgery, *n* (%)	667 (81.6)	1,254 (87.6)	1,118 (64)
Medicine for Older People, *n* (%)	0 (0)	1 (0.1)	6 (0.3)
Included in NELA, *n* (%)	645 (78.9)	842 (58.8)	0 (0.0)
Palliative care received, *n* (%)	79 (9.7)	58 (4.1)	171 (9.8)
Outcomes			
Hospital length of stay, days, median (IQR)	16.8 (9.1–34.3)	8.2 (4.5–16.1)	5.4 (2.7–12)
Mortality			
In-hospital, *n* (%)	132 (16.2)	55 (3.8)	303 (17.3)
30 days, *n* (%)	159 (19.5)	69 (4.8)	392 (22.4)
1 year, *n* (%)	242 (29.6)	158 (11.0)	606 (34.7)
Readmission within 1 year, *n* (%)	293 (35.9)	430 (30.0)	697 (39.9)
Unanticipated ICU admission, *n* (%)	172 (21.1)	125 (8.7)	42 (2.4)
Combined outcome: death or readmission within 1 year, *n* (%)	470 (57.5)	519 (36.2)	1,133 (64.8)

Data are presented as *n* (%) and median (IQR).

Most patients were admitted as an emergency (97.5%) and to a surgical specialty (76.0%). Of the patients admitted under general medicine, the majority received non-operative management. There were few admissions to the Medicines for Older People group, which seems appropriate given the median age of the cohort. Length of hospital stay was shortest in the non-operative group, followed by laparoscopic and then open surgery. Rates of readmission to hospital were similar across treatment groups. Patients who received non-operative management had notably fewer unintended admissions to ICU (2.4%). For cases where surgery was started using an open approach, the rate of unintended admissions to ICU was double that for cases started laparoscopically (21.1% vs 8.7%).

The overall 30-day mortality rate for emergency bowel surgery was comparable to that reported nationally (10.1%).^1^ For patients managed non-operatively, 30-day mortality (22.4%) was not as high as we expected based on rates reported in other studies.^7^ Patients suitable for their surgery to be started laparoscopically were observed to have a much lower 30-day mortality rate than for those who required a laparotomy (4.8% vs 19.5%). 1-year mortality was higher for non-operative management than for surgery (34.7% vs 17.8%).

**Figure 1:**
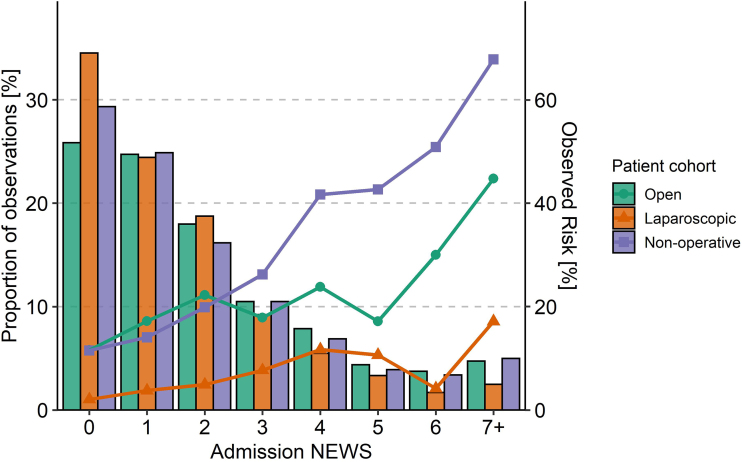
Plot of the cumulative distribution of each NEWS score as a proportion (bars), with the mortality rate for each score plotted over (points), coloured for each treatment group.

In Figure 1, the distribution of each admission NEWS value is displayed as a proportion (bars), with the 30-day mortality rate for each value plotted over (points and line). The overall trend is that mortality increases with a rising admission NEWS. There is, however, a notable jump in mortality at a NEWS threshold of 4 for cases managed non-operatively, which is not observed with surgery. We therefore undertook further analysis to look at only cases with an admission NEWS ≥4; patient demographics and outcomes are summarised for this sub-population in Table 2. Half received non-operative management, with the rest undergoing open or laparoscopic surgery. The 30-day mortality rate increased across all treatment groups but was notably higher for non-operative management compared with surgery (50.3% vs 19.5%).

**Table 2:** Summary of demographic and admission data and outcomes for patients having open surgery, laparoscopy and non-operative treatment with an admission NEWS ≥4

	Open	Laparoscopic	Non-operative
Admissions, *n*	166	183	334
Age, years, median (IQR)	70 (60–78.8)	68 (53–77)	76.5 (66.2–84)
Female, *n* (%)	91 (54.8)	91 (49.7)	189 (56.6)
Type of admission			
Elective, *n* (%)	8 (4.8)	7 (3.8)	0 (0.0)
Emergency, *n* (%)	158 (95.2)	176 (96.2)	334 (100.0)
Admission specialty group			
Medicine, *n* (%)	27 (16.3)	27 (14.8)	146 (43.7)
Surgery, *n* (%)	139 (83.7)	154 (84.2)	188 (56.3)
Medicine for Older People, *n* (%)	0 (0.0)	1 (0.5)	1 (0.3)
Included in NELA, *n* (%)	138 (83.1)	138 (75.4)	0 (0.0)
Palliative care received, *n* (%)	17 (10.2)	16 (8.7)	62 (18.6)
Outcomes			
Hospital length of stay, days, median (IQR)	17.8 (8.4–35.9)	12.7 (6.7–25.7)	7.7 (2.9–16.4)
Mortality			
In-hospital, *n* (%)	40 (24.1)	20 (10.9)	136 (40.7)
30 days, *n* (%)	47 (28.3)	21 (11.5)	168 (50.3)
1 year, *n* (%)	59 (35.5)	37 (20.2)	201 (60.2)
Readmission within 1 year, *n* (%)	66 (39.8)	69 (37.7)	101 (30.2)
Unanticipated ICU admission, *n* (%)	47 (28.3)	31 (16.9)	21 (6.3)
Combined outcome: death or readmission within 1 year, *n* (%)	113 (68.1)	92 (50.3)	273 (81.7)

Data are presented as *n* (%) and median (IQR).

## Discussion

In this article, we present the findings of the largest UK study to date, investigating patients admitted to hospital with an intestinal emergency who received either operative or non-operative management. By conducting a retrospective analysis of EHRs, we have been able to provide a rapid report on this poorly studied population. This builds on the findings of McIlveen *et al* but has also identified some differences in the non-operative group.^7^

Just under half of patients received non-operative management, comparably higher than reported by McIlveen *et al* (44% vs 32%), and the 30-day mortality rate was also significantly lower (22% vs 63%).^7^ We think this difference is partly explained by our broad inclusion criteria (all conditions which may require emergency bowel surgery), whereas McIlveen focused on patients who required (but had been declined) surgery. Thus, we have probably captured data on many patients with an intestinal emergency whose condition could reasonably recover without surgery. Analysis of NEWS scores on admission identified a sudden increase in mortality for the non-operative group at a threshold NEWS of 4, which did not occur with surgery. Examination of this subgroup revealed that 30-day mortality for non-operative treatment was more than double that of the total group (50.3% vs 22.4%) and much more comparable to that reported by McIlveen.^7^

Emergency bowel surgery was routinely started laparoscopically (63%), with a low rate of conversion to open (21.4%). Notable differences were identified between the open and laparoscopic groups, which may partly explain operative decision making. Patients whose surgery was started laparoscopically were observed to be younger, with a much lower unintended ICU admission, postoperative length of stay and mortality than open surgery. In addition, patients requiring primarily open surgery were more likely to be unwell preoperatively with a NEWS score >4 (20.3% vs 12.8%). There is now good evidence to demonstrate that emergency bowel surgery performed laparoscopically confers superior outcomes.^10^ However, in this unadjusted analysis we cannot say with any certainty that differences we have observed are due to surgical approach alone, rather than the other patient and operative factors.

This study is limited by its retrospective observational design, and we recognise this in our methods and interpretation of the results. There will indeed be unmeasured selection bias influencing what treatment patients have received, which we cannot account for using EHRs alone. Furthermore, we do not have data on what actual non-operative treatment patients received, such as nasogastric tube decompression, type of antibiotics or use of parenteral nutrition. There are also no data on why patients did not have surgery, which we feel would be a key outcome for future prospective studies on this topic. Any study using EHRs is also at risk of unknown coding errors occurring. However, our exploratory data analysis of both diagnosis and procedures codes revealed that the depth of coding was extremely thorough. Furthermore, variables such as vital signs, theatre and ICU data are recorded real-time by the clinical team and are likely to be accurate. We also compared data on the operative group with the NELA dataset, demonstrating a high level of agreement.

The strength of this study is that it has provided useful information on a poorly studied patient population and is the largest on this topic to date. The proportion of patients with an intestinal emergency who do not require surgery is greater than we initially expected.^7^ It appears that many respond well to non-operative management with associated short duration of hospital admission. Nonetheless, patients in the non-operative cohort still have a much higher mortality rate than those who underwent surgery. While we have not been able to determine why patients did not undergo surgery, the low rate of admission to ICU for the non-operative group suggests that for many of them, major invasive treatment was not appropriate. We have used elevated NEWS scores to reliably identify patients who were unwell on admission, with revealing findings. Only half of patients in this subgroup who received non-operative treatment were alive at 30 days, with a longer-term mortality rate of 60.2%. This information is useful for clinicians in several ways. First, it supports an early discussion of a ward-based ceiling of care with patients who are not suitable for or do not want emergency bowel surgery, particularly if they are unwell. It also provides an estimated short-term mortality rate of 20% to 50% for discussing non-operative management with patients. The observation that patients who are unwell prior to surgery are likely to have a higher postoperative mortality rate is not a novel finding. However, the clear trend between rising admission NEWS and 30-day mortality may prompt clinicians to proceed with urgent surgery, rather than a trial of non-operative management in conditions that would potentially settle with this.

To better understand the differences in patient characteristics and outcomes between those who do and do not undergo emergency bowel surgery, a further condition-specific analysis is required. While national audits view this population collectively, the actual underlying conditions and surgical treatments are diverse. For example, adhesional small bowel obstruction is the commonest indication for emergency laparotomy.^1^ However, only one-third of cases require surgery, with the rest resolving with nasogastric decompression.^5^ Investigating all treatments together may reveal trends such as the superiority of long-term nasogastric drainage and parenteral nutrition, over high-risk laparotomy in a battle-scarred abdomen. Conversely, the laparoscopic approach could be found to have favourable outcomes in older/frail patients, for whom laparotomy carries increased morbidity and mortality.^11–13^ These examples are theoretical, but serve to illustrate how comparing surgery with non-operative management may identify better treatment options.

A prospective study would capture data on why patients were not offered or declined surgery, whether a trial of non-operative treatment was initiated and investigate the influence (if any) of other factors such as frailty. The decision to proceed with major surgery is a complex one that involves a shared decision-making process with a patient and their family. The second part of the Emergency Laparotomy and Frailty study (ELF-2) is investigating older patients who require but do not undergo emergency bowel surgery, which will also hopefully shed further light on this topic.^14^

In conclusion, this is the second UK study to compare the use of non-operative management with surgery for intestinal emergencies, and the largest to date. A surprisingly high number of patients do not undergo surgery, and further research is required to investigate why this is the case.

## Data Availability

The datasets created for this study are not publicly available as they contain confidential patient data. They are subject to the strict data protection rules outlined in the ethical approval for this study and access is restricted to the relevant members of the research team. Access to these data on request would require the approval from the chief investigator of the study and health research authority.
